# Randomisierte Phase-IIB-Studie zur Protonentherapie vs. intensitätsmodulierte Photonentherapie für lokal fortgeschrittene Ösophaguskarzinome

**DOI:** 10.1007/s00066-020-01714-9

**Published:** 2020-11-30

**Authors:** Mechthild Krause

**Affiliations:** Klinik und Poliklinik für Strahlentherapie und Radioonkologie, Universitätsklinikum Carl Gustav Carus, Technische Universität Dresden, Anstalt des öffentlichen Rechts des Freistaates Sachsen, Fetscherstraße 74, 01307 Dresden, Deutschland

## Ziel der Arbeit

Diese randomisierte Studie vergleicht die Gesamttoxizitätsbelastung (TTB) und das progressionsfreie Überleben (PFS) bei Protonenbestrahlung („proton beam therapy“ [PBT]) und Photonen-Intensitätsmodulierter Strahlentherapie (IMRT) für Patienten mit lokal fortgeschrittenem Ösophaguskarzinom.

## Methode und Patienten

Die Patienten wurden randomisiert mit einer PBT oder IMRT bis zu einer Gesamtdosis von 50,4 Gy bestrahlt, eine Stratifizierung nach Histologie, Resektabilität, Induktionschemotherapie und Tumorstadium erfolgte. Die vorher definierten koprimären Endpunkte waren TTB und PFS. Mit TTB, einem zusammengesetzten Score aus 11 verschiedenen unerwünschten Ereignissen, einschließlich gemeinsamer Toxizitäten sowie postoperativer Komplikationen bei operierten Patienten, wurde das Ausmaß der Nebenwirkungen über 1 Jahr nach der Behandlung quantifiziert. Die Studie erfolgte mit dem Bayes-Design sequenzieller Gruppen („Bayesian sequencial group design“) mit drei geplanten Zwischenanalysen bei 33 %, 50 % und 67 % der erwarteten Rekrutierung, bereinigt um das Follow-up.

## Ergebnisse

Diese im April 2012 begonnene Studie wurde nach der Aktivierung der Phase-III-Nachfolgestudie (NRG-GI006) in Übereinstimmung mit dem „data monitoring and safety board“ in 2019 unmittelbar vor der 67 %-Zwischenanalyse zum Abschluss und zur Analyse genehmigt. Insgesamt 145 Patienten wurden randomisiert (72 IMRT, 73 PBT), die Daten von 107 Patienten (61 IMRT, 46 PBT) waren auswertbar. Die mittlere Nachbeobachtungszeit betrug 44,1 Monate. 51 Patienten (30 IMRT, 21 PBT) wurden einer Ösophagektomie unterzogen. 80 % der PBT-Patienten wurden mit Passive-scattering-Technik behandelt. Der Mittelwert *a posteriori* für die TTB war bei der IMRT 2,3-mal höher (39,9; 95 %-Glaubwürdigkeitsintervall 26,2–54,9) als bei PBT (17,4; 10,5–25,0). Der mittlere Score für postoperative Komplikationen lag 7,6-mal höher für IMRT (19,1; 7,3–32,3) gegenüber PBT (2,5; 0,3–5,2). Die posteriore Wahrscheinlichkeit, dass die mittlere TTB für PBT niedriger war als für IMRT, lag bei 0,9989, was die Abbruchgrenze der Studie von 0,9942 bei der 67 %-Zwischenanalyse überschritt. Die Drei-Jahres-PFS-Rate (50,8 % vs. 51,2 %) und die Drei-Jahres-Gesamtüberlebensrate (44,5 % vs. 51,2 %) waren vergleichbar.

## Schlussfolgerungen der Autoren

Bei lokal fortgeschrittenen Ösophaguskarzinomen reduziert die Protonentherapie das Risiko von Nebenwirkungen und deren Schweregrad im Vergleich zur IMRT, wobei das PFS ähnlich ist.

## Kommentar

Die Protonentherapie hat gegenüber der Photonenstrahlentherapie eine vorteilhaftere physikalische Dosisverteilung mit einem sehr steilen Dosisabfall hinter dem Dosismaximum. Dies führt in der Regel zu einer geringeren Dosisbelastung der das Zielvolumen umgebenden gesunden Gewebe. Für die allermeisten Tumorarten ist bisher jedoch noch nicht erwiesen, dass diese geringere Dosis in Normalgeweben auch zu weniger Nebenwirkungen führt. Deshalb ist in vielen Ländern einschließlich Deutschland die Finanzierung der deutlich teureren Protonentherapie durch die Krankenkassen nicht generell gesichert. Aufgabe der Protonenzentren ist es also, prinzipielle Vergleichsstudien zwischen Protonen und der bestmöglichen Photonenstrahlentherapie durchzuführen, um Patientengruppen zu identifizieren, für die die Protonentherapie tatsächlich medizinisch vorteilhaft ist.

In der vorliegenden Studie [1] ist dies für Patienten mit Ösophaguskarzinom hervorragend gelungen. Das statistische Design erlaubte Zwischenanalysen für den primären Endpunkt und den Abschluss der Studie, wenn die posteriore Wahrscheinlichkeit eines signifikanten Unterschieds zwischen beiden Modalitäten die vordefinierten Stoppkriterien erfüllte. Der Endpunkt „total toxicity burden“ (TTB) bringt den kumulativen Schweregrad multipler unerwünschter Ereignisse zusammen, die nach der Radiochemotherapie (RCT) allein oder perioperativ auftraten, d. h. sowohl RCT-Nebenwirkungen als auch perioperative Komplikationen. Definiert wurde die TTB durch 13 mögliche Fälle von 11 verschiedenen Nebenwirkungen (7 postoperative Komplikationen bis 30 Tage postoperativ: akutes Lungenversagen, Anastomoseninsuffizienz, postoperatives Vorhofflimmern, postoperative Pneumonie, Lungenembolie, Reintubation, Schlaganfall; 6 potenziell rezidivierende Toxizitäten, die bis zu 12 Monate nach Randomisation auftraten: Vorhofflimmern, Myokardinfarkt, Perikarderguss, Pleuraerguss, Pneumonie, Pneumonitis). Die Schweregrade dieser Ereignisse wurden nach CTCAE v 4.0 definiert. Die Schwere der Toxizitäten wurde mit einem vorher durch eine multidisziplinäre Gruppe aus Radioonkologen, medizinischen und chirurgischen Onkologen mit einem numerischen Gewicht (0–100) versehen. Die numerischen Werte beschreiben das vom Arzt wahrgenommene relative Ausmaß des medizinischen Schadens, den ein Patient erleidet. Zusätzlich wurden das tumorspezifische Therapieergebnis, Lymphozytopenie als Zeichen der Immunsuppression und die von den Patienten berichtete Lebensqualität ausgewertet. Die relativ hohe Zahl nicht auswertbarer Patienten in der Protonengruppe begründete sich in der Regel mit einer Nichtfinanzierung der Protonentherapie durch die jeweilige Krankenkasse, in der Photonengruppe mit dem Patientenwunsch, mit der Protonentherapie behandelt zu werden.

Für die primäre Analyse der TTB zum Zeitpunkt des Datenschlusses betrug die posteriore Wahrscheinlichkeit, dass die mittlere TTB für die Protonentherapie niedriger ist als für die IMRT, 0,9989 und übertraf damit die vorher festgelegte Stoppgrenze des Studiendesigns von 0,9942 bei der 67 %-Zwischenanalyse. Der posteriore Mittelwert der TTB war 2,3-mal höher für IMRT (39,9; 95 % höchstes posteriores Dichteintervall, 26,2–54,9) im Vergleich zur Protonentherapie (17,4; 10,5–25,0). Bei der Auswertung der operierten Patienten war der durchschnittliche Wert der postoperativen Komplikationen sogar 7,6-mal höher nach IMRT (19,1; 7,3–32,3) vs. Protonentherapie (2,5; 0,3–5,2; *p*-Wert 0,018 und 0,02). Der Unterschied begründete sich in erster Linie in kardiopulmonalen Toxizitäten und postoperativen Komplikationen. Hierzu wurde fast gleichzeitig noch eine separate Analyse publiziert [2], zu der ebenfalls ein Literaturkommentar geschrieben wurde. Progressionsfreies Überleben und Gesamtüberleben waren nicht unterschiedlich in den beiden Therapiearmen. In der Lebensqualität ergab sich kein signifikanter Unterschied. In der Protonentherapiegruppe traten signifikant weniger hochgradige Lymphozytopenien auf. In der Protonentherapiegruppe war bei vergleichbarer Größe der Zielvolumina die Lungendosis signifikant niedriger (V5, 41,4 % vs. 19,7 %; V20, 13,6 % vs. 8,4 %; mittlere Lungendosis, 8,4 vs. 4,8 Gy; *P* = 0,001 für alle), ebenso die Herzdosis (19,8 vs. 11,3 Gy; *P* = 0,001) und die Leberdosis (12,1 vs. 2,4 Gy; *P* = 0,001).

Wegen dieser klaren Befunde ist der Stellenwert der Studie besonders hoch. Bisher lagen ja nur ausschließlich vergleichende dosimetrische Studien vor, nicht jedoch klinisch vergleichende prospektive Studien zur Protonen- im Vergleich zur Photonentherapie beim Ösophaguskarzinom. Diese Studie liefert die erste randomisierte Evidenz für den Nutzen der Protonentherapie in der onkologischen Behandlung und legt die Vermutung nahe, dass die Protonentherapie die weniger nebenwirkungsträchtige Modalität ist als die IMRT bei einem ähnlichen progressionsfreien Überleben.

Die Methode der Studiendurchführung erscheint uns hervorragend. Der Unterschied zwischen den beiden Modalitäten war wahrscheinlich überwiegend durch den Unterschied bei den postoperativen Komplikationen bedingt, was auch vorherige retrospektive Auswertungen nahegelegt hatten [3, 4]. Der fehlende Unterschied in der Lebensqualität lässt sich am ehesten damit begründen, dass die allgemeine und nicht die krankheitsspezifische Lebensqualität abgefragt wurde, dafür war die Studie auch nicht gepowert. Die geringeren Lymphozytopenieraten nach Protonentherapie lassen sich durch das geringere bestrahlte Volumen erklären. Ein Zusammenhang zwischen Bestrahlungstechnik und Überleben besteht übrigens in der bisherigen Nachbeobachtungszeit nicht.

Gewisse Schwächen der Studie bestehen darin, dass der Endpunkt TTB bisher nicht validiert ist, dass relativ viele Patienten nicht auswertbar waren aufgrund von Finanzierungsproblemen der Protonentherapie und dass es sich um eine unizentrische Studie handelt. Eine multizentrische Phase-III-Studie wurde initiiert, um diese Unsicherheiten zu beseitigen.

## Fazit

Die Studie zeigt erstmalig in einem direkten randomisierten Vergleich eine Überlegenheit der Protonentherapie gegenüber Photonen bei einer Tumorart. Der Unterschied scheint insbesondere bei den postoperativen Komplikationen groß zu sein. Insbesondere solche Patienten, bei denen neoadjuvant ein relativ hohes Herz- und/oder Lungenvolumen mitbestrahlt werden muss, sollten über die Möglichkeit einer Protonentherapie beraten werden.

*Mechthild Krause, Dresden*
